# Bioenergetic Adaptations in Chemoresistant Ovarian Cancer Cells

**DOI:** 10.1038/s41598-017-09206-0

**Published:** 2017-08-18

**Authors:** Sajad Dar, Jasdeep Chhina, Ismail Mert, Dhananjay Chitale, Thomas Buekers, Hareena Kaur, Shailendra Giri, Adnan Munkarah, Ramandeep Rattan

**Affiliations:** 1 Division of Gynecology Oncology, Department of Women’s Health Services, Henry Ford Health System, Detroit, MI 48202 USA; 2 Department of Obstetrics and Gynecology, Wayne State School of Medicine, Detroit, MI 48202 USA; 3 Department of Pathology, Henry Ford Health System, Detroit, MI 48202 USA; 4 Department of Neurology, Henry Ford Health System, Detroit, MI 48202 USA

## Abstract

Earlier investigations have revealed that tumor cells undergo metabolic reprogramming and mainly derive their cellular energy from aerobic glycolysis rather than oxidative phosphorylation even in the presence of oxygen. However, recent studies have shown that certain cancer cells display increased oxidative phosphorylation or high metabolically active phenotype. Cellular bioenergetic profiling of 13 established and 12 patient derived ovarian cancer cell lines revealed significant bioenergetics diversity. The bioenergetics phenotype of ovarian cancer cell lines correlated with functional phenotypes of doubling time and oxidative stress. Interestingly, chemosensitive cancer cell lines (A2780 and PEO1) displayed a glycolytic phenotype while their chemoresistant counterparts (C200 and PEO4) exhibited a high metabolically active phenotype with the ability to switch between oxidative phosphorylation or glycolysis. The chemosensitive cancer cells could not survive glucose deprivation, while the chemoresistant cells displayed adaptability. In the patient derived ovarian cancer cells, a similar correlation was observed between a high metabolically active phenotype and chemoresistance. Thus, ovarian cancer cells seem to display heterogeneity in using glycolysis or oxidative phosphorylation as an energy source. The flexibility in using different energy pathways may indicate a survival adaptation to achieve a higher ‘cellular fitness’ that may be also associated with chemoresistance.

## Introduction

Human cells meet energy needs through glycolysis and oxidative phosphorylation (OXPHOS) pathways. Although both pathways produce the adenosine triphosphate (ATP) required by the cell to continue its growth and regulation processes, OXPHOS produces higher levels of ATP. Cancer cells have been shown to devise a shift in energy production from OXPHOS to glycolysis, even in the presence of oxygen; this phenomenon is commonly known as the Warburg Effect^1, 2^ and is marked by an increased glucose uptake and lactate production. This reliance of cancer cells on glycolysis compared to non-cancerous cells has been attributed to their need to sustain increased proliferation rate and evade death inducing signals^3^. The alterations and adaptions of the glycolytic pathway have been shown to occur at multiple levels including overexpression of glycolytic enzymes, defects in the OXPHOS machinery or oncogenic transformations^4, 5^.

Increased glucose consumption in cancer cells is devoted to lactate conversion and is uncoupled from oxidative metabolism^6^. Glycolysis and lactate are not only required as fuel sources, but the glycolytic breakdown of glucose also produces various intermediate metabolites that are utilized in anabolic pathways namely pentose phosphate pathway, serine and triacylglycerol biosynthesis, de novo synthesis of nucleotides, amino acids, and lipids^7^. Thus, glycolysis is essential for both energy production and synthesis of numerous cellular components required for growth and proliferation. Furthermore, aerobic glycolysis may also occur in the stromal compartment surrounding the tumor, thus providing additional metabolites to the cancer cells^8^. This dependency of some cancer cells on glycolysis has provided a new potential therapeutic target. Glycolysis inhibitors have been shown to exhibit antitumor effects in various cancers when used alone and in combination with other modalities and are being pursued in clinical trials^9–11^.

The reliance on glycolysis in some cancer cells has been previously attributed to impaired mitochondria^12, 13^. However, current data have shown that mitochondria are functional in many cancer cells^14^. Furthermore, recent work reveals that cancer cells are not solely dependent on glycolysis for their energy requirement but also derive energy from mitochondrial respiration^15–17^. Invasive migratory ovarian cancer cells and ovarian cancer stem cells have been shown to display metabolic heterogeneity and prefer OXPHOS^6, 18–21^. The cellular function, fuel type and microenvironment cues, and the interplay between these play a central regulating role in energy metabolism in cancer cells^22–24^.

Understanding the bioenergetic phenotype of cancer cells can open a new horizon in cancer treatment for most malignancies including ovarian cancer^25^. Bioenergetic profiling of ovarian cancer cells may be utilized in investigating therapeutic options, better characterize different histological subtypes and stem cells^26, 27^. In the present study, we characterize the bioenergetic profiles of 13 established and 12 patient derived ovarian cancer cells and show the prevalence of metabolic heterogeneity in use of energy pathways. We also investigate the association between the high metabolically active phenotype of ovarian cancer cells and their chemoresistance.

## Results

### Bioenergetic profile of ovarian cancer cell lines

**Figure 1:**
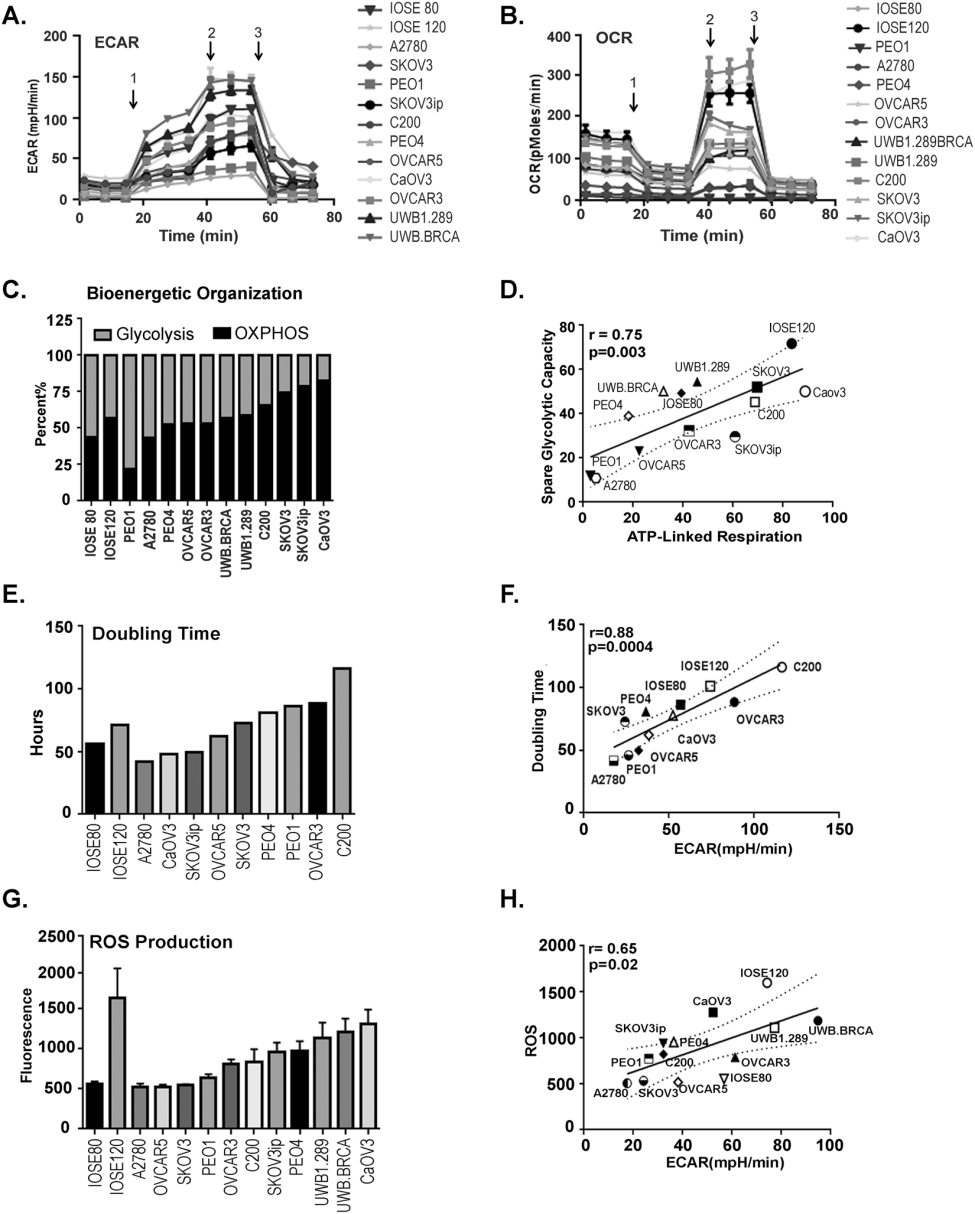
Bioenergetics profile of ovarian cancer cell lines. (**A**) Extracellular acidification rate (ECAR) was measured in various ovarian cancer cell lines as described in the Materials and Methods when challenged by (1) glucose (fuel for glycolysis), (2) oligomycin (an ATP synthase blocker) and (3) 2DG (an inhibitor of glycolysis). (**B**) Oxygen consumption rate (OCR) was measured in various ovarian cancer cell lines as described in the Materials and Methods by a cell mitochondrial stress test when challenged by (1) oligomycin (an ATP synthase inhibitor), (2) FCCP (an electron transport chain uncoupler) and (3) rotenone (an inhibitor of electron transport chain). (**C**) Bioenergetics organization was obtained as the percent ratio of basal OCR and ECAR. (**D**) Positive correlation between ATP-linked respiration (representing OCR) and spare glycolytic capacity (representing glycolytic ability). (**E**) Doubling time of various ovarian cancer cell lines obtained from a 7-day growth curve of each cell line. (**F**) Positive correlation between doubling time and ECAR. (**G**) Basal reactive oxygen species (ROS) generation in various ovarian cancer cell lines measured by using DCFDA dye at 30 minutes. (**H**) Positive correlation between ROS levels and ECAR. All Seahorse experiments (Seahorse Biosciences, Boston, MA) were performed for a minimum of 3 times in triplicates. Abbreviations: ATP: adenosine triphosphatase; 2DG: 2-deoxyglucose; FCCP: carbonylcyanide-p-trifluoromethoxyphenyl hydrazine; DCFDA: 2′,7′-dichlorofluorescin diacetate.

We analyzed the bioenergetic profile of 11 ovarian cancer cell lines (A2780, PEO1, OVCAR3, OVCAR5, C200, PEO4, UWB.126, UWB.126 BRCA, SKOV3, SKOV3IP and CaOV3) and 2 immortalized ovarian surface epithelium cell lines (IOSE 80 and IOSE120) as described in the Materials and Methods. Glycolytic function was estimated as an indirect measurement of the extracellular acidification rate (ECAR)^28, 29^. The various ovarian cancer cell lines, including the 2 IOSEs, showed a diverse glycolytic function exhibiting diverse responses to the various glycolytic flux tests (Fig. 1A). Cells were incubated in glucose free media to allow for 3 readings, followed by addition of glucose (injection 1), to which all cell lines showed an increase in ECAR. Addition of oligomycin (injection 2) to inhibit mitochondrial ATP production resulted in a further increase in glycolysis as an alternative energy producing step in most cell lines. Lastly, addition of 2DG (injection 3) inhibited all glycolysis activity in the cells showing a drop in ECAR (Fig. 1A). Mitochondrial function of all the ovarian cancer cell lines was determined through oxygen consumption rate (OCR) of cells in culture media as an indirect measure of mitochondrial respiration or OXPHOS^26, 28–30^. Similar to the glycolytic profile, a wide OXPHOS function was observed within our panel of cell lines (Fig. 1B). Baseline OCR measurements were followed by the addition of oligomycin (injection 1) that inhibited mitochondrial ATP synthase, causing a decrease in OCR. This was followed by addition of carbonilcyanide p-triflouromethoxyphenylhydrazone (FCCP; injection 2), which causes uncoupling of mitochondrial OX-PHOS and induced maximal respiration. Lastly a combination of rotenone and antimycin D (injection 3) was added to inhibit complex I of the mitochondrial respiration chain (Fig. 1B). Individual basal ECAR, glycolytic capacity, glycolytic reserve and basal OCR, maximum respiration and respiratory reserve are presented in Supplementary Fig. S1. To get an overall picture of the bioenergetics organization of the cells, a ratio of basal glycolysis vs. basal OXPHOS was generated. For this, bioenergetics profiles obtained through the cell mitochondrial stress media data were used, in which all components are available to the cell via the complete media. Basal OCR and ECAR were averaged using the first 3 readings and a percent ratio was generated, which reflects the baseline energy pathway preference of each particular cell line (Fig. 1C). Most of the cell lines suggested an equal use of both glycolysis and OXPHOS pathways. PEO1 and A2780 profile indicated preference of the glycolysis pathway, whereas SKOV3, SKOV3-IP, and Ca-OV3 appeared to favor the OXPHOS pathway. A positive correlation was seen between ATP-linked and spare respiratory capacity (correlation coefficient of r = 0.7509, p = 0.0031), or glycolytic reserve, demonstrating cells with lower ATP-linked respiration favor glycolysis (Fig. 1D)^31^.

The preference of a particular energy pathway has been linked to fulfilling the cancer cell’s particular growth or functional requirement. Increased glycolysis has been associated with sustenance of faster proliferation rate^32^. To examine if a faster proliferation rate is associated with glycolysis in our panel of ovarian cancer cell lines, we correlated the doubling time of the cell lines with basal ECAR, representing the glycolysis rate of these cells lines. A proliferation curve over 5 days was generated for A2780, PEO1, SKOV3, PEO4, OVCAR5, IOSE120, IOSE 80, CaOV3, C200 and OVCAR3 to calculate the doubling time (Fig. 1E). The doubling time of the various cell lines significantly correlated with ECAR (p = 0.0004, r = 0.88; Fig. 1F), indicating that the proliferation rate of the ovarian cancer cell lines and the preference for glycolysis has a positive relationship. Another function that correlated with glycolysis is the production of reactive oxygen species (ROS). Cancer cells have been shown to prefer glycolysis to maintain low oxidative stress, since OXPHOS results in generation of free oxygen radicles^6^. We also observed a lower ROS generation in the cells with higher glycolysis rates (Fig. 1G) which correlated positively with basal ECAR (p = 0.02, r = 0.65; Fig. 1H). Overall, various ovarian cancer cell lines, including IOSE80 and IOSE120, exhibit significant diversity in their bioenergetics profiles, indicating the metabolic individuality of each cell line. Additionally, the bioenergetics profiles of cancer cells positively correlated with their functional phenotype.

### Bioenergetic organization of chemosensitive and chemoresistant ovarian cancer cells

**Figure 2:**
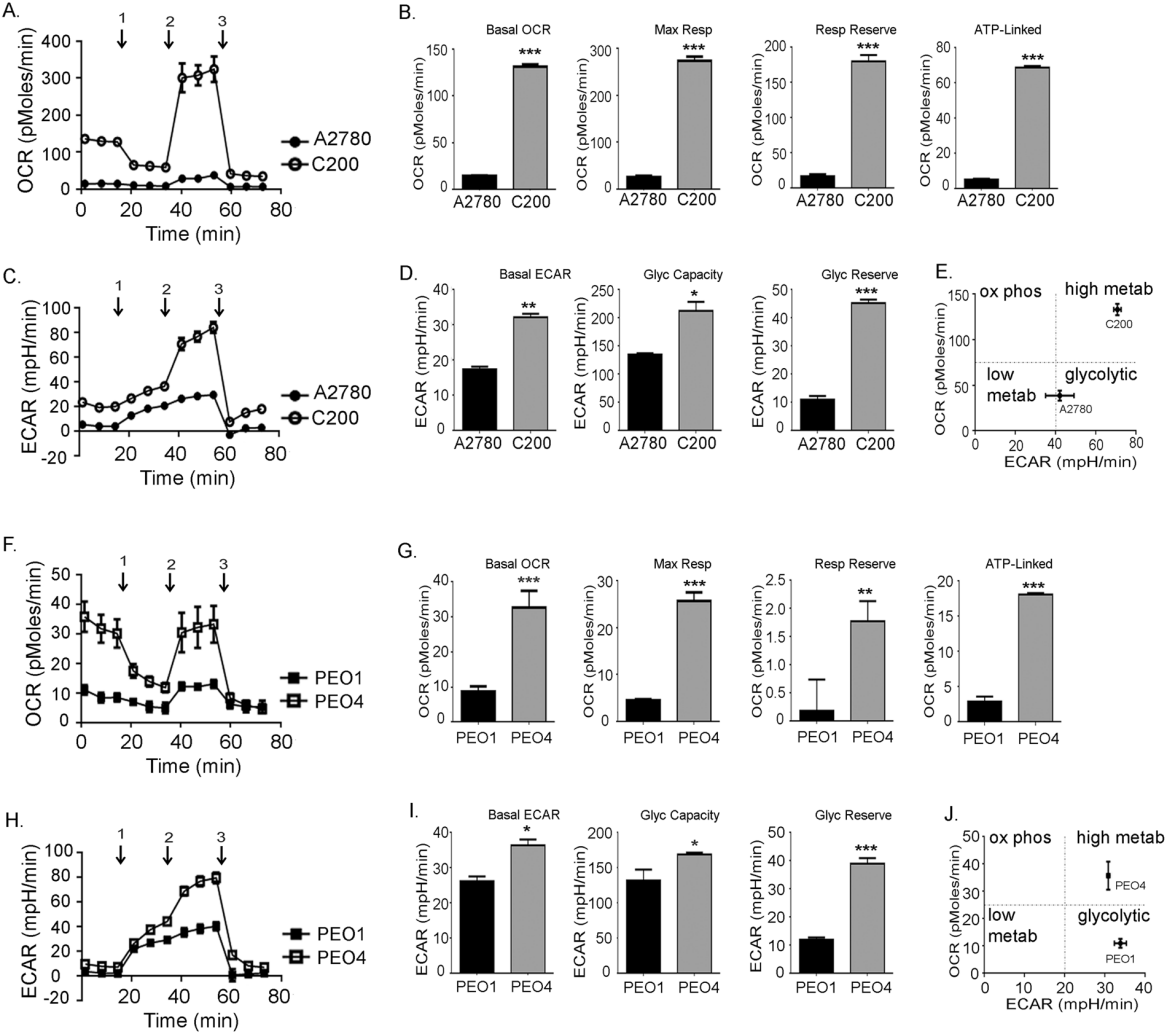
Bioenergetics organization of chemosensitive and chemoresistant ovarian cancer cells. (**A**) Oxygen consumption rate (OCR) was measured in chemosensitive A2780 and resistant C200 isogenic cell lines and (**F**) chemosensitive PEO1 and resistant PEO4 isogenic cell lines by a cell mitochondrial stress test when challenged by (1) oligomycin (an ATP synthase inhibitor), (2) FCCP (an electron transport chain uncoupler) and (3) rotenone (an inhibitor of electron transport chain). (**B**,**G**) Basal OCR represents the mitochondrial respiration at resting state, the maximum respiration indicates the cellular reaction to an increased ATP demand using FCCP mitochondrial uncoupler, and respiratory reserve and ATP-linked respiration indicate greater capacity to utilize mitochondria. These were calculated as described in the Materials and Methods. ***p < 0.001, **p < 0.01 chemoresistant cells (C200 and PEO4 compared to respective chemosensitive cells [A2780 and PEO1]). Extracellular acidification rate (ECAR) was measured in (**C**) chemosensitive A2780 and chemoresistant C200 isogenic cell lines and (**H**) chemosensitive PEO1 and resistant PEO4 isogenic cell lines when challenged by (1) glucose (fuel for glycolysis), (2) oligomycin (an ATP synthase blocker) and (3) 2DG (an inhibitor of glycolysis). (**D**,**I**) Basal ECAR represents the glycolysis measure at resting state, glycolytic capacity represents the total ability of the cell to perform glycolysis (resting state and enforced combined) and glycolytic reserve defines the capacity to increase glycolysis when mitochondria is compromised, were calculated as described in the methods. ***p < 0.001, **p < 0.01, *p < 0.05 chemoresistant cells (C200 and PEO4 compared to respective chemosensitive cells [A2780 and PEO1]). (**E**,**J**) Ratio of OCR: ECAR quadrant showing the bioenergetics phenotype of each cell line. All Seahorse experiments (Seahorse Biosciences, Boston, MA) were performed for a minimum of 5 times in triplicates. Abbreviations: ATP: adenosine triphosphatase; 2DG: 2-deoxyglucose; FCCP: carbonylcyanide-p-trifluoromethoxyphenyl hydrazine.

An interesting observation that became apparent from the bioenergetics of various ovarian cancer cell lines was the differential bioenergetics profiles of chemosensitive and chemoresistant cells. Our panel of ovarian cancer cell lines included chemosensitive ovarian cancer cell lines: A2780 and PEO1 and their respective chemoresistant counterparts C200 and PEO4. The chemosensitive A2780 and PEO1 had a significantly lower OXPHOS (Fig. 2A and F) as well as glycolysis function (Fig. 2C and H) compared to their respective chemoresistant counterparts C200 and PEO4. This was also reflected in lower basal ECAR values that defined the glycolysis measure at resting state. Glycolytic capacity is the total ability of the cell to perform glycolysis (resting state and enforced combined) and glycolytic reserve that defines the capacity to increase glycolysis when mitochondria is compromised by oligomycin (Fig. 2D and I). Along with increased glycolysis, the chemoresistant cells also performed higher OXPHOS, as reflected in their basal OCR measurement, maximum respiration that is an indication of the cellular reaction to an increased ATP demand using FCCP mitochondrial uncoupler, respiratory reserve and ATP-linked respiration that indicate greater utilization of mitochondria (Fig. 2B and G). Individually, the chemosensitive cells had increased glycolysis than OXPHOS function, which classified them as glycolytic while the chemoresistant cell lines showed higher glycolysis as well as higher OXPHOS, which classified them as having a highly metabolic active phenotype (Fig. 2E and J). Together it can be summarized that chemoresistant and chemosensitive ovarian cancer cells may exhibit distinct bioenergetics profiles where the chemosensitive cells rely more on glycolysis and the chemoresistant cells orchestrate both energy pathways equally.

### Glycolytic and mitochondrial gene expression

**Figure 3:**
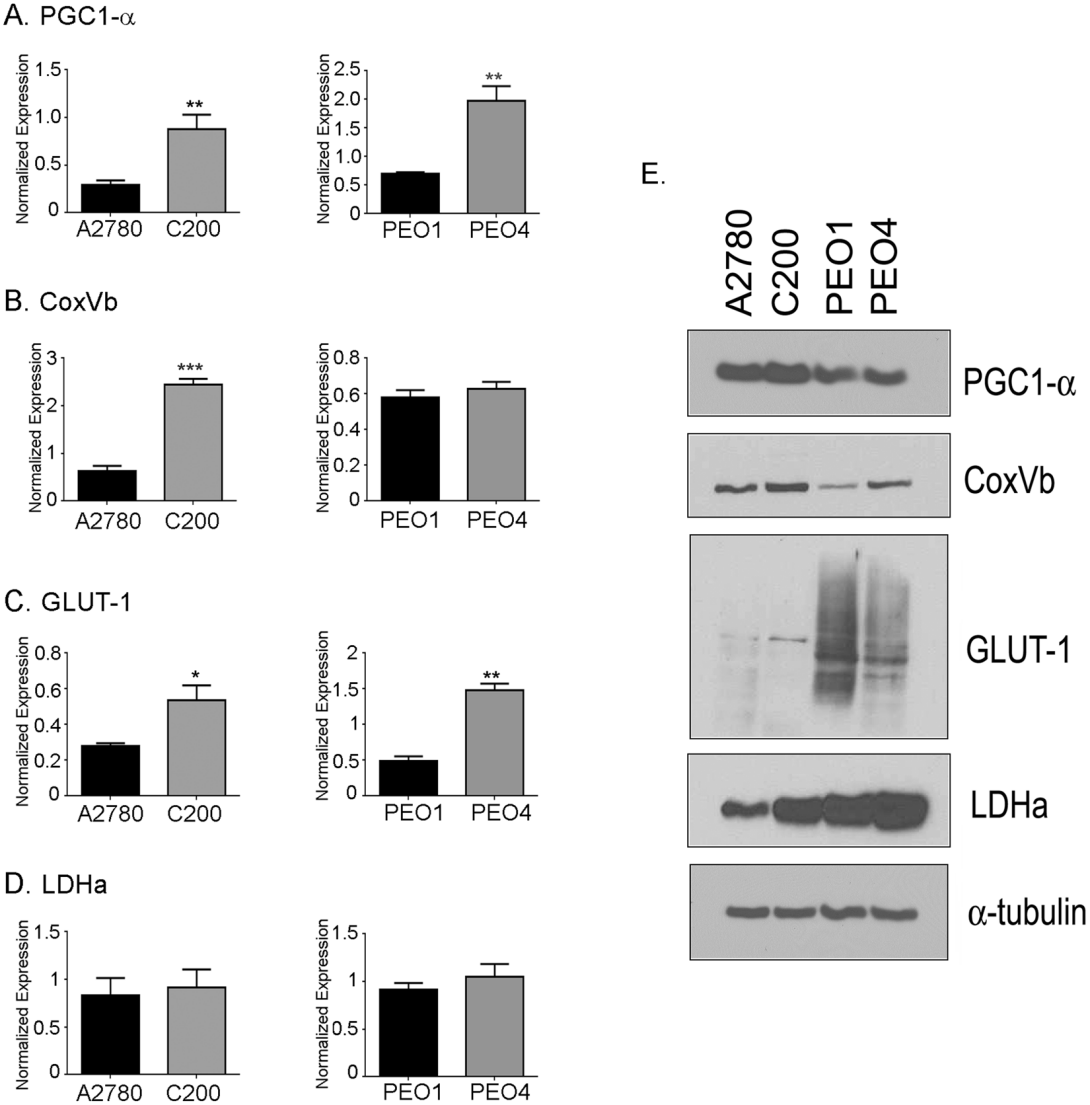
Glycolytic and mitochondrial gene expression. Extracted mRNA from cells was used to synthesize cDNA and expression of (**A**) PGC1α, (**B**) CoxVb, (**C**) GLUT1 and (**D**) LDHa was determined by real-time polymerase chain reaction (RT-PCR). The RT-PCR was ran in triplicates and is representative of 2 individual runs. ***p < 0.001, **p < 0.01, *p < 0.05 chemoresistant cells (C200 and PEO4 compared to respective chemosensitive cells A2780 and PEO1). (**E**) The protein extracted from cells was subjected to western blot and immuno-blotted for expression of PGC1α, CoxVb, GLUT1 and LDHa. Alpha- tublin acted as the loading control. The cropped blots are representative of 2 individually performed experiments. Abbreviations: PGC1α: peroxisome proliferator-activated receptor gamma coactivator 1-alpha; CoxVb: cytochrome c oxidase subunit 5B; GLUT1: glucose transporter 1 and LDHa: lactate dehydrogenase-A.

To extend the bioenergetics profile of the chemosensitive and chemoresistant cells, we investigated the expression of key genes in glycolysis and OXPHOS at the RNA and protein level. The chemoresistant C200 and PEO4 cells showed increased expression of the mitochondrial gene PGC1α both at RNA and protein levels (Fig. 3A and E: top panel). The mitochondrial enzyme COXVb was found to be significantly increased only in C200 cells at the mRNA level (Fig. 3B) but appeared to be increased at protein level in both C200 and PEO4 (Fig. 3E: second panel). The glycolysis linked gene GLUT1, responsible for glucose uptake, was higher in chemoresistant C200 cells at mRNA and protein compared to sensitive A2780 cells. While the chemoresistant PEO4 showed significant difference in the GLUT1 gene at the RNA level, the GLUT1 protein expression showed the opposite, with the sensitive PEO1 cells showing an increased GLUT1 expression (Fig. 3C and E: third panel). The lactate dehydrogenase-α expression was unchanged at mRNA level between the 2 subsets, however, the chemoresistant cells showed an increased protein expression compared to the sensitive cells (Fig. 3D and E: fourth panel). Beta-tubulin acted as the loading control. Thus, the expression profiles of the genes were mostly consistent with the different bioenergetics phenotype of the glycolytic sensitive and the highly metabolic active chemoresistant cells that exhibited increased expression of both OXPHOS and glycolytic genes.

### Chemoresistant ovarian cancer cells have an increased mitochondrial function

**Figure 4:**
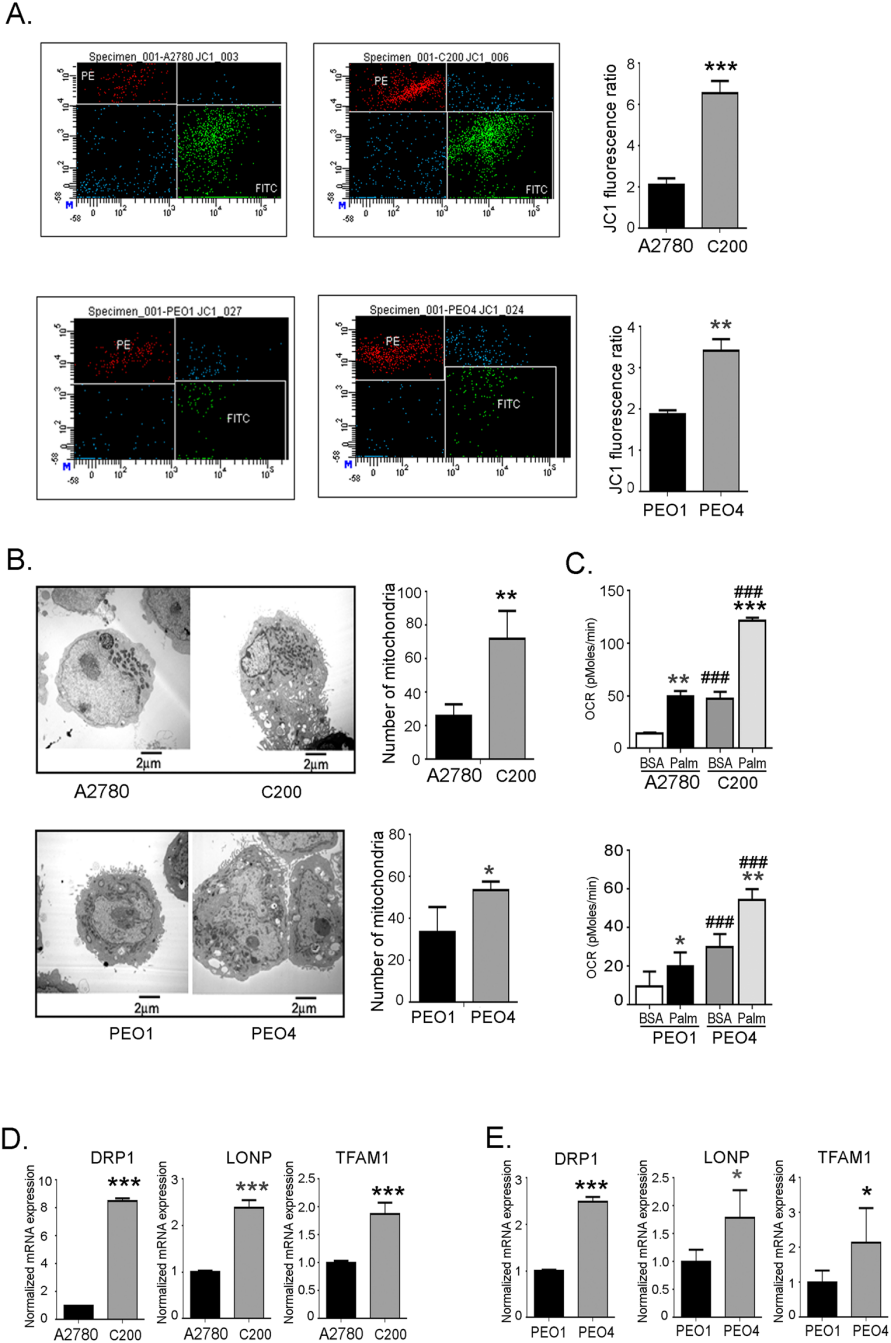
Chemoresistant ovarian cancer cells have increased mitochondrial function. (**A**) Mitochondrial potential of the cells was measured by JC-1 staining. The green fluorescence indicates de-energized mitochondria and the red fluorescence indicates energized mitochondria. Red to green fluorescence intensity ratio indicated increased mitochondrial potential and is represented as a bar graph. The experiment was performed in triplicates and replicated twice. ***p < 0.001, **p < 0.01, chemoresistant cells (C200 and PEO4 compared to respective chemosensitive cells [A2780 and PEO1]). (**B**) Representative electron microscope pictures of mitochondria. Cells were washed, fixed, embedded in Spurr’s resin and 90 nm sections were placed on 200 mesh copper grids and then examined under Philips Transmission Electron Microscope 208. Mitochondrial count was performed by counting mitochondria per high power field from 15 fields per cell line. **p < 0.01, *p < 0.05 chemoresistant cells (C200 and PEO1 compared to respective chemosensitive cells [A2780 and PEO1]). (**C**) Palmitate-linked OCR representing fatty acid oxidation. Bovine Serum Albumin (BSA) is the vehicle control for palmitate. ***p < 0.001, **p < 0.01, *p < 0.05 palmitate compared to BSA in respective cell line. ###p < 0.001, resistant cells (C200 and PEO4) compared to sensitive cells (A2780 and PEO1). (**D**,**E**) Extracted mRNA from cells was used to synthesize cDNA and expression of mitochondrial biogenesis genes was estimated by RT-PCR. The RT-PCR polymerase chain reaction was ran in triplicates and is representative of 2 individual runs. ***p < 0.001, *p < 0.05 chemoresistant cells (C200 and PEO4 compared to respective chemosensitive cells [A2780 and PEO1]). Abbreviations: DRP1: dynamin-related protein; LONP: Lon protease homolog, mitochondrial; TFAM1: mitochondrial transcription factor A, JC-1: 5,5′,6,6′-tetrachloro-1,1′,3,3′-tetraethylbenzimi-dazolylcarbocyanine iodide.

To further address the distinction between sensitive and chemoresistant cells, we looked at their mitochondrial function. Mitochondrial membrane potential, measured by JC-1 staining, showed increased mitochondrial membrane potential in the chemoresistant C200 and PEO4 cells compared to the chemosensitive A2780 and PEO1 cells (Fig. 4A), indicating hyperpolarization of mitochondria in chemoresistant cells, which may lead to an increased bioenergetic capacity. An approximate 2-fold increase in the ROS levels of the chemoresistant cells (C200, PEO4) compared to the sensitive cells (A2780, PEO1) was also observed earlier (Fig. 1G). The increase in ROS levels also indicates increased mitochondrial metabolism^33^.

To further see if the increased mitochondrial function reflects the mitochondria number in these cells, transmission electron microscopy pictographs were observed to study the mitochondria. C200 and PEO4 cells showed significantly higher mitochondrial density compared to A2780 and PEO1, respectively, which was reflected in mitochondrial number counts (Fig. 4B). In accordance, the chemoresistant cells showed a higher fatty acid oxidation rate at the basal level and also when measured by palmitate-induced OXPHOS, compared to sensitive cells (Fig. 4C). The palmitate-induced OXPHOS as an indicator of fatty acid oxidation was confirmed by using etomoxir, the fatty acid oxidation inhibitor as a control (Supplementary Fig. S2). As further validation, we observed the genes involved in maintaining mitochondria number and integrity. Dynamin-1-like protein, that regulates mitochondria fission; Lon protease homolog, that acts as mitochondrial protein control; and mitochondrial transcription factor A, a key activator of mitochondrial transcription as well as a participant in mitochondrial genome replication, were assessed by quantitative RT-PCR. Both C200 and PEO4 showed an increased expression of all 3 mitochondria related genes compared to A2780 and PEO1, respectively (Fig. 4D and E). Overall, the chemoresistant cells are indicated to have an increased number of mitochondria that may function more robustly compared to mitochondria of the chemosensitive ovarian cancer cells.

### Chemosensitive ovarian cancer cells are more sensitive to glucose deprivation

**Figure 5:**
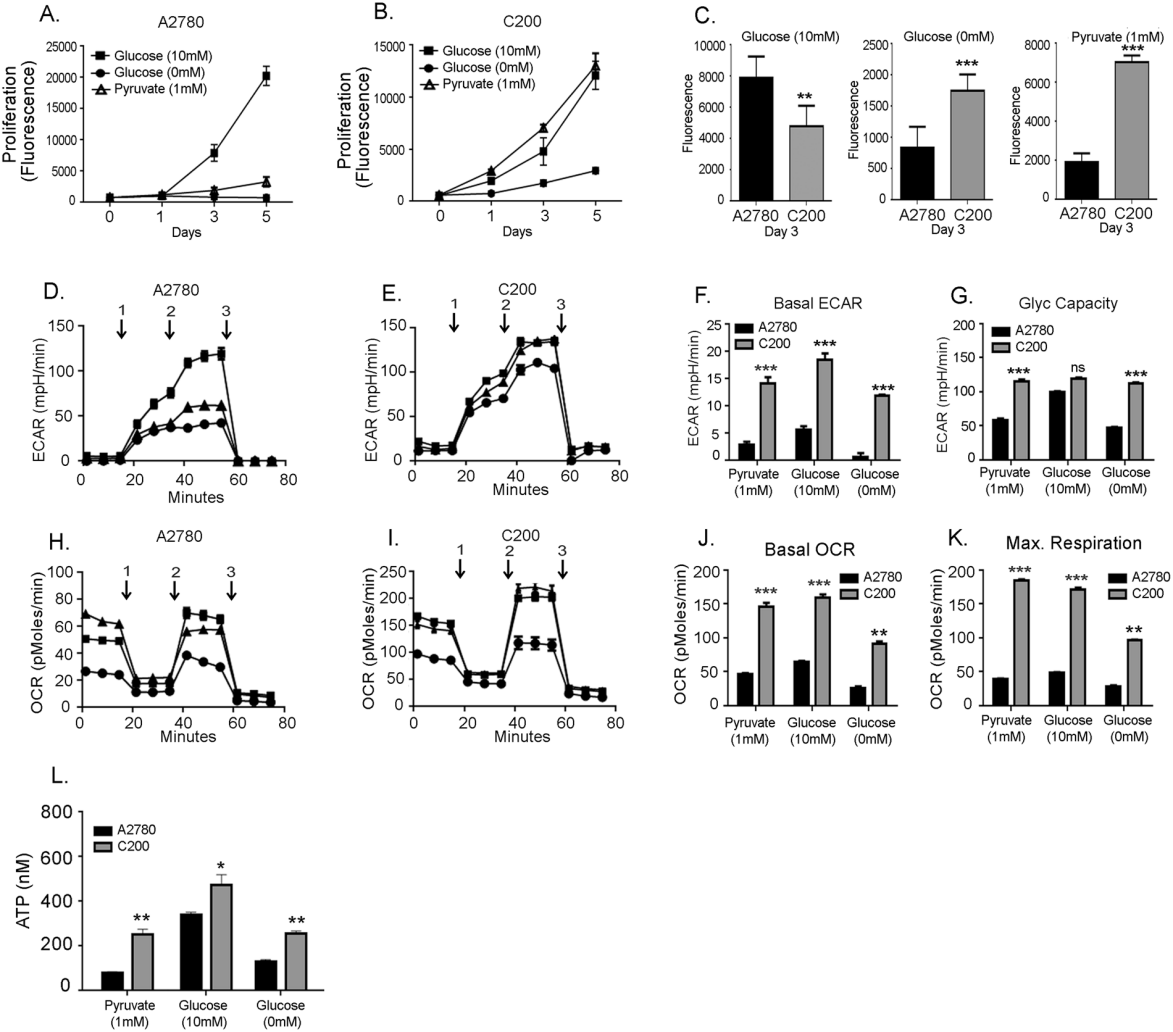
Chemosensitive ovarian cancer cells are more sensitive to glucose deprivation. (**A**) A2780 and (**B**) C200 cells were cultured in glucose free (0mM glucose) Roswell Park Memorial Institute 1640 media containing 5% dialyzed fetal bovine serum or supplemented with 10 mM glucose or 1 mM pyruvate. Proliferation of cells was measured using CYQUANT® NF cell Proliferation Assay by measuring fluorescence intensity at different time points as indicated. (**C**) Bar graph representation of the cell proliferation under various conditions at day 3. The experiment was performed in triplicates and repeated twice. ***p < 0.001, **p < 0.01 C200 compared to A2780. Under similar conditions, at 48 h, an equal number of cells were plated in multi-well Seahorse Biosciences XF96 plate (Boston, MA) for measurement of ECAR (**D**,**E**) and OCR (**H**,**I**). For ECAR, injections 1, 2, 3 represents glucose, oligomycin and 2DG respectively. For OCR, injections 1,2,3 represent oligomycin, FCCP and rotenone respectively. Basal ECAR (**F**), glycolytic capacity (**G**), basal OCR (**J**) and maximum respiration (**K**) were calculated as described in the Materials and Methods and are represented as bar graphs. The experiment was performed in triplicates and repeated twice. ***p < 0.001, **p < 0.01 C200 compared to A2780. (**I**) Under similar conditions ATP production was measured by a Biovision fluorescence assay kit. ***p < 0.001, **p < 0.01, C200 compared to A2780.

Glucose deprivation has been shown to make tumor cells more susceptible to apoptosis alone and in combination with other chemotherapeutic stress^34, 35^. Since the chemosensitive cells indicated a glycolytic profile, we sought to confirm if these cells would be more susceptible to glucose deprivation than the chemoresistant cells. The chemosensitive cells proliferated faster than the chemoresistant C200 cells under normal media conditions containing 10 mM glucose (Fig. 5A–C). Under glucose deprivation, A2780 cells demonstrated nearly complete growth inhibition over 5 days of culture (Fig. 5A). Moreover, supplementation with sodium pyruvate to fuel OXPHOS did not show any significant increase in growth rate over time (Fig. 5A and C). Conversely, C200 cells appeared relatively resistant to glucose deprivation and proliferated at a faster rate compared to A2780 (Fig. 5B and C), although the proliferation was significantly less as compared to that in glucose containing media (Fig. 5B). On supplementation with sodium pyruvate, C200 were able to thrive and proliferate almost equally at the same rate as in the glucose containing media (Fig. 5B).

Bioenergetic analysis under similar conditions was performed to understand the energy pathway utilization by chemosensitive and chemoresistant cells. Compared to cells grown in glucose supplemented media, A2780 cells cultured in glucose free media showed a 90% decrease in basal ECAR (Fig. 5D and F) and an almost 60% decrease in glycolytic capacity (Fig. 5G) in response to stimulation with glucose and inhibition of OXPHOS with oligomycin. C200 cells on the other hand showed only a 25–30% decrease in basal ECAR (Fig. 5E and F) and a ~10% increase in glycolytic capacity (Fig. 5G) indicating their better ability to manage glucose deprivation by switching to OXPHOS. This was demonstrated by their increased OCR (~4-fold) compared to A2780 (Fig. 5H–J) and also by their increased sensitivity to FCCP (~3-fold), demonstrating their highly increased maximum respiratory capacity (Fig. 5K) under all conditions. This was further confirmed by estimating the levels of cellular ATP under similar conditions, which showed the ability of C200 to sustain higher ATP levels in glucose free conditions and also their potential to increase ATP production more efficiently than A2780 when supplemented with glucose or sodium pyruvate (Fig. 5L).

Thus, the chemoresistant ovarian cancer cells appear to be more resilient to glucose deprivation and are able to use either energy pathway under glucose stress conditions.

### Resistant ovarian cancer C200 cells have the ability to switch between energy states

**Figure 6:**
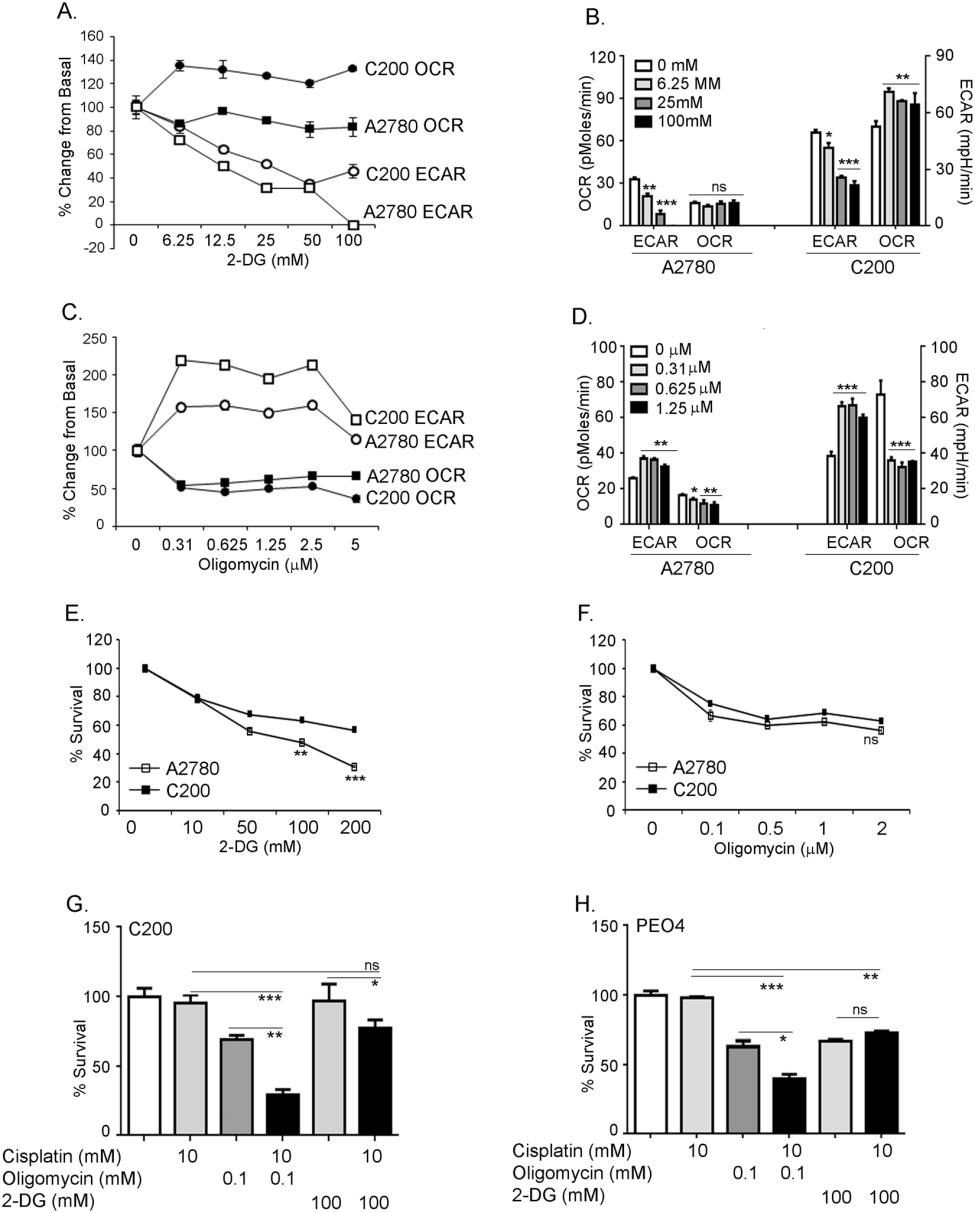
Resistant ovarian cancer C200 cells have the ability to switch between energy states. (**A**) Equal number of cells were plated in multi-well Seahorse Biosciences XF96 plate (Boston, MA), and after calibration time indicated doses of 2-deoxyglucose (2DG) were injected and measurements of OCR and ECAR were recorded in triplicates. (**B**) Bar graph representation of the dose-dependent effect on ECAR and OCR after 2DG inhibition of glycolysis. ***p < 0.001, **p < 0.01, *p < 0.05 2DG treated compared to untreated. (**C**) Equal number of cells were plated in multi-well Seahorse Biosciences XF96 plate, and after calibration time indicated doses of oligomycin were injected and measurements of OCR and ECAR were recorded in triplicates. (**D**) Bar graph representation of the dose-dependent effect on ECAR and OCR after oligomycin inhibition of mitochondrial function. ***p < 0.001, **p < 0.01, *p < 0.05 oligomycin treated compared to untreated. All Seahorse experiments were performed in triplicates and replicated twice. A2780 and C200 were plated at 4000 cells per well in 96 wells and treated with indicated concentrations of (**E**) 2DG or (**F**) oligomycin and live cells were measured at 48 h using Promega Cell Viability Assay. The data is represented as percent survival compared to untreated control cells, which were taken as 100% live cells. **p < 0.01, *p < 0.05 C200 compared to A2780. The experiment was performed in triplicates thrice. Chemoresistant C200 (**G**) and PEO4 (**H**) cells were treated with cisplatin in the presence or absence of 2DG or oligomycin and live cells were measured at 48 h using Promega Cell Viability Assay. The data is represented as percent survival compared to untreated control cells, which were taken as 100% live cells. The cell survival studies were carried out in triplicates and replicated thrice. ***p < 0.001, **p < 0.01, *p < 0.05 combination compared to individual treatments.

To further confirm the resilient nature of chemoresistant cells and their ability to switch between the energy pathways, we evaluated the bioenergetics of A2780 and C200 cells in the presence or absence of 2-deoxyglucose (2DG), the glycolysis inhibitor, and oligomycin A (OM), an inhibitor of ATP synthase. A2780 and C200 cells were exposed to increasing doses of 2DG and OM to simultaneously measure ECAR and OCR using the Seahorse XF analyzer (Seahorse Biosciences, Boston, MA). Injecting 2DG at different concentrations showed a dose dependent decline in ECAR in both the cell lines; however, OCR was maintained at a similar level as the untreated cells in A2780 (Fig. 6A and B), suggesting their impaired or insufficient OXPHOS capacity to meet the increased energy demand. On the other hand, OCR increased significantly in C200 cells in response to increasing 2DG concentrations (Fig. 6A and B), demonstrating competence of C200 to use other substrates for energy production like pyruvate or glutamine via the tricarboxylic acid cycle and OXPHOS. In the presence of increasing doses of OM, the OCR rate declined in both the cell lines, with a more pronounced effect in the C200 cells (Fig. 6C and D). As a consequence of OCR suppression by OM, C200 cells accelerated glycolytic flux as indicated by their increased ECAR and A2780 were also able to increase ECAR, but to a lesser extent (Fig. 6C and D). Thus, the chemoresistant C200 cells with higher respiration levels show larger gains in glycolysis flux upon OM treatment and larger OXPHOS compensation on 2DG glycolysis inhibition, indicating an increased ability in utilizing the energy pathways.

To investigate if the bioenergetics flexibility translates to susceptibility to metabolic inhibitors, we measured the cell viability under 2DG and OM treatments. A2780 cells displayed a higher dose dependent decrease in cell proliferation with 2DG (~50% decrease at 200 mM) than C200 (~30% decrease at 200 mM), reiterating that glycolysis being the main source of sustenance for A2780 cells (Fig. 6E). Oligomycin treatment did not show any significant difference in response between the 2 cell lines, although A2780 also displayed a trend of decreased viability under OM (Fig. 6F). This could be suggestive of C200’s ability to sustain survival under OM by using glycolysis at an increased capacity (Fig. 6C,D) compared to A2780. We next investigated whether 2DG or OM would be a better approach to increase the sensitivity of the chemoresistant cells to cisplatin. Chemoresistant C200 and PEO4 cells were treated with cisplatin in combination with 2DG (10 mM) or OM (0.1 µM) and cell viability was analyzed at 48 h. Treatment with combination of OM and cisplatin was more cytotoxic (25% viability in C200 and 40% viability in PEO4) compared to 2DG and cisplatin combination treatment (75% viability in C200 and 60% viability in PEO4) in both the chemoresistant cell lines compared to cisplatin alone (Fig. 6G and H). Thus combining mitochondrial inhibitors with platinum chemotherapy may be a better intervention for targeting chemoresistant ovarian cancer cells.

### Cisplatin induces a highly active metabolic state

**Figure 7:**
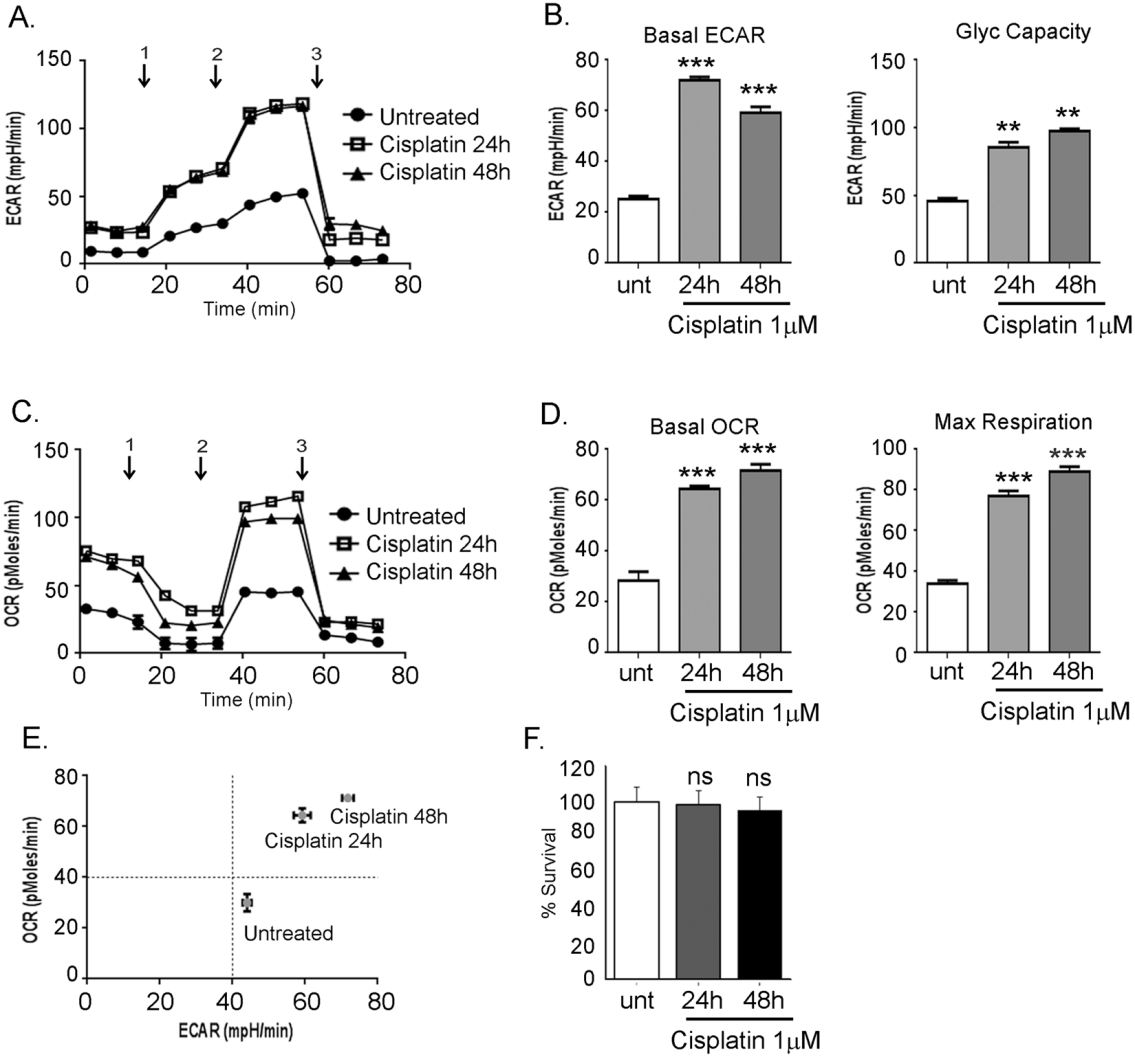
Cisplatin induces a highly active metabolic state in chemosensitive cells. A2780 chemosensitive cells were treated with a non-toxic low dose of cisplatin for 24 and 48 hours and then subjected to measurement of (**A**) ECAR profile after (1) glucose (fuel for glycolysis), (2) oligomycin (an ATP synthase blocker) and (3) 2DG (an inhibitor of glycolysis) injections (**C**) OCR profile measured under similar conditions after (1) oligomycin (ATP synthase inhibitor), (2) FCCP (an electron transport chain uncoupler) and (3) rotenone (an inhibitor of electron transport chain) injections. (**B**) Basal glycolysis and glycolytic capacity is increased by cisplatin exposure. (**D**) Basal mitochondrial respiration and maximum respiration is increased by cisplatin exposure. (**E**) OCR: ECAR ration indicate a shift in bioenergetics phenotype from glycolytic to highly active metabolism after cisplatin treatment. (**F**) Cisplatin (1 uM) is non-toxic at the concentration used. All Seahorse experiments were carried out in triplicates and replicated thrice. ***p < 0.001, **p < 0.01, NS: non-significant cisplatin treated compared to untreated. Abbreviations: ATP: adenosine triphosphatase; 2DG: 2-deoxyglucose; FCCP: carbonylcyanide-p-trifluoromethoxyphenyl hydrazine. OCR: Oxygen Consumption Rate; ECAR: Extracellular Acidification Rate.

Our data strongly suggests that the chemoresistant cells lines are highly metabolically active, which enables them to utilize both energy pathways effectively for survival. An important question we wanted to address was if chemotherapy treatment can induce metabolic change in the chemosensitive cell line that may act as a harbinger of acquiring chemoresistance. The sensitive A2780 cells were exposed to low dose of cisplatin (1 uM) and the bioenergetics profile was evaluated post 24 and 48 h. Cisplatin treatment resulted in both increased ECAR (Fig. 7A) and OCR (Fig. 7C) response. Increased glycolysis under cisplatin treatment was reflected in the increased basal ECAR and glycolytic capacity (Fig. 7B). Similarly, increased basal OCR and maximum respiration (Fig. 7D) reflected an increased OXPHOS response. This resulted in cisplatin treatment shifting the glycolytic phenotype of sensitive A2780 cells towards a highly active metabolic phenotype (Fig. 7E) similar to that observed in chemoresistant ovarian cell lines (Fig. 2E and J). Figure 7F shows that the cisplatin (1 uM) dose used was non-cytotoxic at 48 h. To test if the metabolic shift is platinum specific or a general phenomenon with standard chemotherapy, we also treated the A2780 cells with paclitaxel (1 and 2 nM) and performed the bioenergetics response. Paclitaxel treatment did not result in any increment in ECAR or OCR response (Supplementary Fig. S3), indicating that the metabolic shift observed is unique to platinum exposure. Thus, cisplatin exposure may be able to induce bioenergetic phenotype changes from glycolysis to being metabolically active.

### Profiling of patient derived ovarian cancer cells

**Figure 8:**
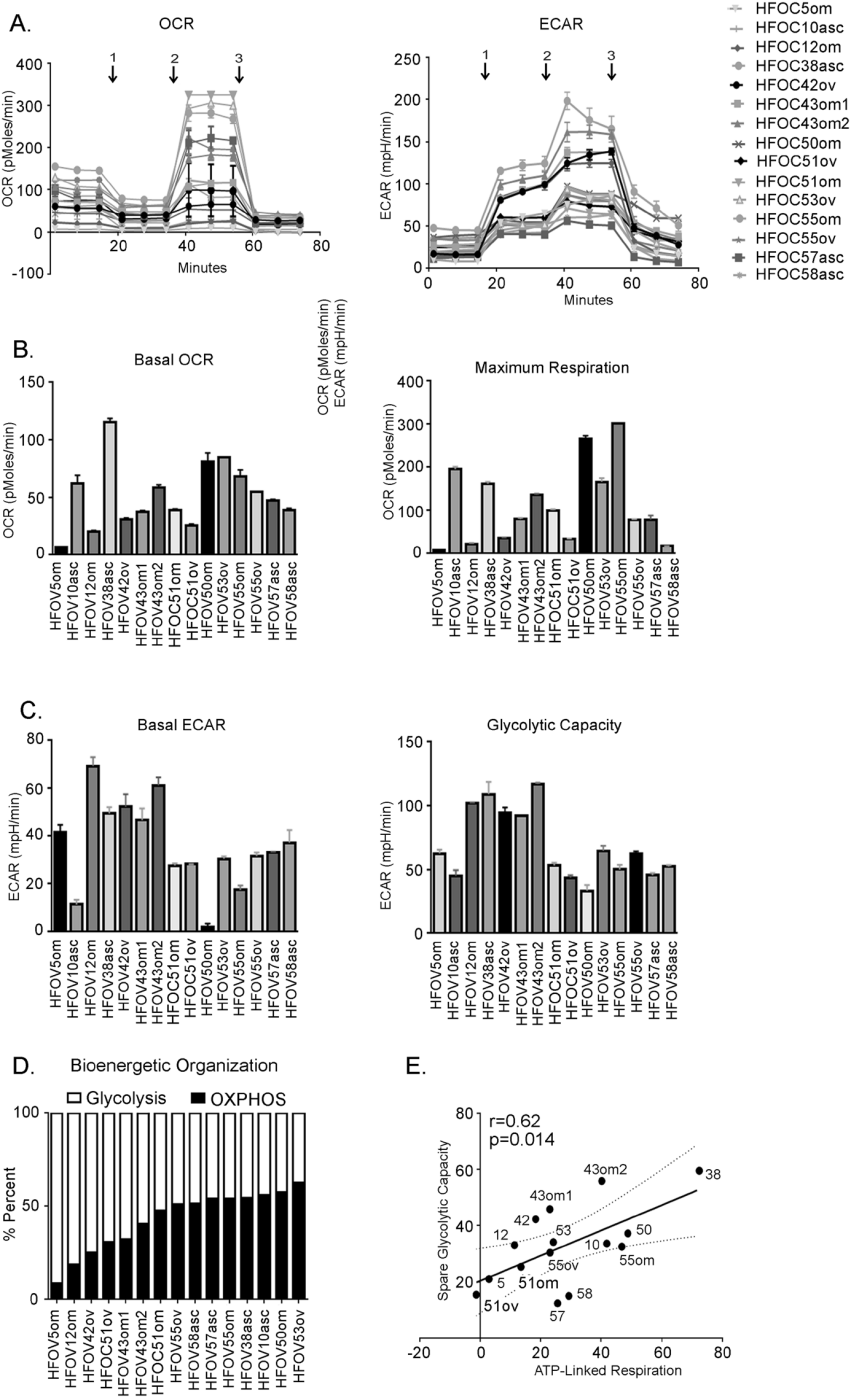
Profiling of patient derived ovarian cancer cells. Ovarian cancer cells were isolated from either tumor specimens or ascites samples of naïve patients diagnosed with ovarian cancer as described in the Materials and Methods. An equal number of cells were plated in multi-well Seahorse Biosciences XF96 plate (Boston, MA) and (**A**) oxygen consumption rate (OCR) and ECAR was measured under challenge by various metabolic regulators as described earlier. The ECAR profile injections were (1) glucose (fuel for glycolysis), (2) oligomycin (an ATP synthase blocker) and (3) 2DG (an inhibitor of glycolysis). OCR profile injections were (1) oligomycin (an ATP synthase inhibitor), (2) FCCP (an electron transport chain uncoupler) and (3) rotenone (an inhibitor of electron transport chain) injections. (**B**) Range of basal OCR and maximum capacity in the patient derived ovarian cancer cells. (**C**) Range of basal ECAR and glycolytic capacity in the patient derived ovarian cancer cells. (**D**) Ratio of basal ECAR and OCR represented as percent to represent the bioenergetic preference of a cell. (**E**) Positive correlation between ATP-linked respiration and spare glycolytic capacity. Seahorse experiments were replicated twice in triplicates. Abbreviations: ATP: adenosine triphosphatase; 2DG: 2-deoxyglucose; FCCP: carbonylcyanide-p-trifluoromethoxyphenyl hydrazine; OCR: Oxygen Consumption Rate; ECAR: Extracellular Acidification Rate.

**Figure 9:**
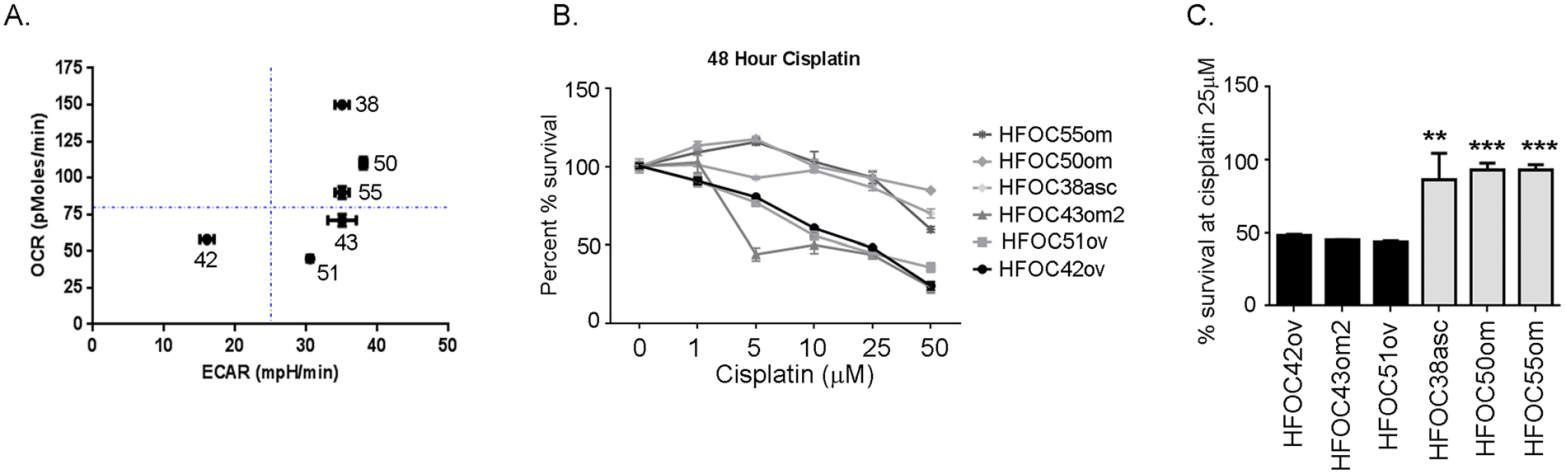
Patient derived ovarian cancer cells show similar relationship between bioenergetics phenotype and chemosensitivity. (**A**) Ratio of OCR: ECAR plotted as a quadrant showing the bioenergetics phenotype of selected patient derived cells. (**B**) Selected patient derived cells that presented as glycolytic or low metabolic activity (HFOC42, HFOC 49 and HFOC 43) and those that presented as high metabolically active (HFOC 38, HFOC 50 and HFOC 55) were subjected to cisplatin treatment at indicated concentrations and cell survival was determined by Promega Cell Viability Assay at 48 h. Data is presented as percent against control taken as 100% viable. (**C**) Bar representation of the percent surviving cells compared to respective control cells at 48 h under 25 uM cisplatin exposure. ***p < 0.001, **p < 0.01, cisplatin treated compared to untreated. Abbreviations: OCR: Oxygen Consumption Rate; ECAR: Extracellular Acidification Rate; HFOC: Henry Ford Ovarian Cancer cell line.

To investigate if similar variation in energy utilization can be validated in the patient population, we isolated 15 epithelial ovarian tumor cells from either the tumor tissue or ascites of chemonaive ovarian cancer patients. The cell lines were designated as Henry Ford Ovarian Cancer (HFOCnumber) and with the site of origin (om: omentum; asc: ascites; and ov: ovary). The ovarian tumor cells from the patients presented a similar picture of variable bioenergetics in response to the various stresses provided during the mitochondrial respiration test and the glycolytic stress test (Fig. 8A). Basal OCR and maximum respiration (Fig. 8B) showed large diversity across the various patients irrespective of whether the cells were isolated from the tumor tissue or ascites as seen in the cancer cell lines. Similar diversity was also seen in the basal glycolysis rate ECAR and glycolytic capacity (Fig. 8C). Basal bioenergetics ratios suggested a variable energy phenotype, with some cells being clearly glycolytic, while most appear to use both energy pathways equally (Fig. 8D), very similar to that observed in the cell line profile (Fig. 1C). The ATP-linked respiration correlated significantly (r = 0.62; p = 0.01) with glycolytic spare capacity (Fig. 8E), indicating that cells with higher ATP were utilizing more OXPHOS and those with low ATP were performing glycolysis. Further, we selected the tumor cells that displayed a low metabolic or glycolytic (HFOV 42, 43, and 51) phenotype and few that displayed a metabolically active phenotype (HFOV 38, 50, and 55), to investigate if their chemoresponse to cisplatin was reflective of their bioenergetics (Fig. 9A). A cisplatin dose response suggests that the highly metabolic active patient derived ovarian tumor cells were indeed resistant to cisplatin when compared to the low metabolic/glycolytic patient derived ovarian tumor cells (Fig. 9B and C). Thus, ovarian cancer cells isolated from patients exhibited variable cellular bioenergetics phenotypes that may offer an insight on their chemoresponse, in a manner very similar to that observed in the various ovarian cancer cell lines.

## Discussion

Warburg was the first to report that cancer cells exhibit enhanced glycolysis and reduced OXPHOS^2^. It was initially thought that the reliance of cancer cells on glycolysis was due to impairment in the OXPHOS mechanisms. Current evidence shows that OXPHOS pathways are intact in most cancer cells and the increased glycolysis is the result of an intricate interplay between oncogenes and the tumor microenvironment^36, 37^. The discovery of altered cellular metabolism in cancer cells has fueled the interest in understanding the specific metabolic attributes of those cancer cells^38, 39^.

We characterized the bioenergetics profile of a panel of ovarian cancer cell lines and showed the existence of pronounced heterogeneity in their bioenergetics requirements with ATP-linked respiration positively correlating with spare glycolytic capacity in these cell lines. While few cancer cell lines were glycolytic in nature (PEO1 and A2780), most displayed a variable ability to utilize and perform glycolysis and OX-PHOS, similar to that seen in immortalized ovarian surface epithelial cells. Bioenergetic characterization of ovarian cancer cells isolated from the tumor or ascites of chemonaïve ovarian cancer patients also revealed a similar picture, confirming the presence of a metabolic heterogeneity in ovarian cancer. Variability in the bioenergetics profiles has been described in breast, sarcoma and pancreatic cancer cell lines^31, 40^. A recent analysis by Dier *et al*. of the bioenergetic dynamics and histology of 8 ovarian cancer cell lines also showed a diversity in the energy dynamics, similar to our findings. Furthermore, they suggested a possible association between histologic tumor subtype and its bioenergy profile^26^. Fabian *et al*. compared 2 ovarian cell lines with less glycolytic (IGROV1) or more glycolytic (OC316) phenotypes and demonstrated that their metabolic phenotype is maintained *in vivo* ^41^.

Increased glycolysis seems to be a survival mechanism that promotes tumor progression and metastasis. Glycolysis is also a source of various macromolecules that are used by cancer cells for the synthesis of essential cell components such as proteins, nucleotides and lipids, and would enable them to proliferate rapidly^7^. In fact, glycolytic cancer cells, including ovarian, have been shown to be more invasive and metastatic^42–44^. We found that basal glycolysis rates significantly correlated with increased proliferation rates, indicating that cells that proliferated faster used more glycolysis. Dier at al. showed that the ability to survive under anchorage-independent conditions, a prerequisite for metastatic spread, was higher in glycolytic ovarian cells compared to those with a more aerobic phenotype. Use of the glycolysis pathway allows for less production of ROS, by avoiding excess ROS generation from oxidative respiration^6^. ROS has been associated with development of cellular senescence, which can cause growth arrest and apoptosis^45–47^. We observed a significant positive correlation between ROS levels and glycolysis.

An interesting observation in a few publications is that the ovarian cell lines with high glycolysis also showed high OXPHOS and associated gene expression, indicating that most of the ovarian cancer cell lines favor a highly metabolic phenotype^26, 41^. Recently, ovarian cancer cell migration was shown to be fueled by pyruvate, implicating mitochondrial activity in the metastatic process^19^. Other investigations have shown that some invasive ovarian cancer cells favor the tricarboxylic acid cycle by increased utilization of glutamine, indicating a role for OXPHOS^18^. The data regarding the bioenergetics of ovarian cancer stem cells are more varied. Pasto *et al*. showed that CD44^+^CD117^+^ ovarian cancer stem cells isolated from epithelial ovarian cancer patients showed both a high glucose uptake and a high OXPHOS phenotype (active metabolic phenotype), which was associated with their increased ability to survive under glucose free conditions^21^. On the other hand, Alvero *et al*. reported that CD44^+^MyD88^+^ cancer stem cells possessed a mainly glycolytic phenotype and proposed that maintenance therapy with glycolytic inhibitors could be beneficial to improve patient’s survival^48^. Mouse ovarian tumor initiating cells (putative cancer stem cells) were shown to possess a highly adaptable metabolic phenotype, whereby they could utilize either glycolysis or OXPHOS under appropriate stress^49^. There is an emerging school of thought that most ovarian cancer cells may utilize either an increased glycolysis or OXPHOS and that such flexibility confers a higher level of ‘cellular fitness’^19, 26, 41^. This concept has also been observed and defined in other studies^50^.

Our work also demonstrated that the 2 chemoresistant ovarian cancer cell lines (C200 and PEO4)^51, 52^ exhibited a high metabolically active phenotype as compared to their respective sensitive counterparts (A2780 and PEO1). Further they demonstrated a metabolic flexibility in using glycolysis or OXPHOS, especially when compromised. These chemoresistant ovarian cancer cells were able to maintain their energy production compared to when they were deprived of glucose, a characteristic not seen in the chemosensitive cells. Pasto *et al*. reported a similar observation where they could predict clinical responsiveness to chemotherapy based on the glucose dependency of neoplastic cells harvested from ovarian cancer patients^53^. Thus, cancer cells with a high metabolically active phenotype may be able to survive a chemotherapy insult better than those with either a glycolytic or an aerobic phenotype. The cancer cells seemed to switch to a high metabolically active phenotype, as they acquire chemoresistance.

Others have demonstrated that ovarian cancer stem cells possessed a similar metabolic phenotype and displayed similar flexibility^21, 49^. In addition, patient-derived ovarian cancer cells that displayed a high metabolically active phenotype were more platinum resistant than the cells that were purely glycolytic. While at this juncture, we cannot comment if the metabolic adaptation in chemoresistant cells is a driver or an outcome event of chemoresistance, our data strongly suggests that metabolic changes occur as a result of chemotherapy drugs.

Our data highlights the diversity in the bioenergetics requirements of the ovarian cancer cells both at the cell line and patient derived tumor cell level. This metabolic diversity could be the result of regulation of metabolism at multiple levels that involves multiple oncogenes, tumor suppressor genes and various microenvironment players. Most ovarian cancer cells maintain both glycolytic and OXPHOS pathways and exhibit a high metabolically active phenotype. While this phenotype may not drive a higher proliferation rate, it appears to make them survive anchorage independent conditions, become invasive, overcome metabolic inhibitors^26, 54^ and possibly promote chemoresistance. Thus, the bioenergetics profile may convey to us the status of the cellular fitness or survival ability of an ovarian cancer cell.

## Material and Methods

### Cell Lines

Eleven ovarian cancer cell lines (A2780, PEO1, OVCAR3, OVCAR5, C200, PEO4, UWB.126, UWB.126 BRCA, SKOV3, SKOV3IP and CaOV3) and 2 immortalized ovarian surface epithelial cell lines (IOSE80 and IOSE120) were maintained in their respective media^55, 56^. A2780 and C200 were a kind gift from the Fox Chase Institute (Dr. Tom Hamilton). OVCAR3, OVCAR5, UWB.126, UWB.126 BRCA, SKOV3 and CaOV3 were purchased from ATCC (Manassas, VA). Transformed ovarian surface epithelial cell lines IOSE 80 and 120 were obtained from the Canadian Ovarian Tissue Bank (University of British Columbia, Vancouver; Canada). PEO1 and PEO4 were purchased from Sigma (St. Louis, MO).

### Bioenergetic Measurements

In order to measure glycolysis and mitochondrial respiration, in real time, the Seahorse XFe Extracellular Flux Analyzer (Seahorse Biosciences) was used, as described previously^29, 55^. The outputs were recorded as ECAR and OCR, respectively. In brief, cells were grown, maintained and treated in their respective media. On the day of the experiment, 5 × 10^4^ cells were plated in a Cell-Tak (Corning, Fisher Scientific) coated XF96 96-well microplate using 175 µL of cell mitochondrial stress media containing 3% glucose or glycolytic stress test media without glucose. After plating, cells were incubated for 1 h at 37 °C without carbon dioxide to allow for cells to reach the ideal pH and temperature conditions required for the assay. The machine ran a 3 minute mix and 3 minute read cycle which generated OCR and ECAR readings. During the assay, various compounds were injected via ports to see their effects on glycolysis and mitochondrial respiration. Three ECAR measurements were recorded after each port injection starting with glucose (10 mM), followed by oligomycin (2 µM), and lastly 2DG (100 mM). OCR measurements were recorded similarly except the port injection started with oligomycin (1 µM), followed by FCCP (250 nM), and lastly a combination of antimycin A and rotenone (both 1 µM). The assay plates included control blank wells containing only media to which various reagents were added similar to experimental wells. The blanks were automatically subtracted by the instrument software. The measurements were normalized with cell number. Basal OCR was taken as the average of the first 3 measurements subtracted by the OCR measurements after rotenone and antimycin D injections. It indicates the baseline of respiration of the cell in the presence of glucose. Basal ECAR was calculated as ECAR_Glucose_ − ECAR_2DG_ when given glucose as fuel. ECAR_Oligo_ − ECAR_2DG_ indicates the maximal glycolysis that can be performed by the cell when mitochondrial respiration is inhibited by oligomycin, Glycolytic reserve (ECAR _glucose_ −ECAR_Oligo_) represents the dependency of cancer cells on glycolysis as a means of energy production. Maximum respiration was calculated as OCR_FCCP_ − OCR_Anti-Rot_ and respiratory reserve was calculated by subtraction of the basal OCR from the maximum respiration^26, 30^.

For the glucose deprivation experiment, cells were cultured in glucose free Roswell Park Memorial Institute 1640 media containing 5% dialyzed fetal bovine serum supplemented with (i) no glucose (glucose−), (ii) 10 mM glucose (glucose+), and (iii) 1 mM sodium pyruvate for 48 h. Equal number of cells (50,000 per well) was then plated in multi-well Seahorse Biosciences XF96 plate prior to measurement of ECAR and OCR. Viability was measured using the CYQUANT® NF Cell Proliferation Assay kit (Thermo Fisher Scientific, Waltham, MA).

Due to the expected 20% variation observed from experiment to experiment, each Seahorse experiment was performed for a minimum of 5 times or more. Data presented is average of 3 individually performed experiments.

### Real time semi-quantitative polymerase chain reaction

Cells were plated and total RNA was extracted using TRIzol Reagent (Life Technologies, Waltham, MA) as indicated by the manufacturer which was followed by cDNA synthesis, as described previously^57^. All primers were purchased from RealtimePrimers.com (Elkins Park, PA). PP1A was used as a housekeeping gene, and polymerase chain reactions were performed using Bio-Rad reagents in Bio-Rad CFX96TM real time instrument (Hercules, CA).

### Electron microscopy

Cells were washed and fixed in Trump’s fixative (1% glutaraldehyde and 4% formaldehyde in 0.1 M phosphate buffer, pH 7.2) for 24 h. Cell pellet was embedded in Spurr’s resin and thin (90 nm) sections were cut on a Reichert Ultracut E ultramicrotome (Leica, Buffalo Grove, IL) and placed on 200 mesh copper grids. Pictures were taken using Philips Transmission Electron Microscope 208 at the Henry Ford Hospital Electron Microscopy Core^58^.

### Doubling Time and proliferation

To define the growth rate of each cell line by doubling time, cell proliferation assays were done over a period of 5 days. There were 20,000 cells/well plated in a 24-well plate per cell line in their respective medias. Viable cell counts were performed by Trypan blue counts on days 0, 1, 3, and 5^29^. Doubling time was calculated using online resources^59, 60^.

### CellTiter-Fluor™ Cell Viability Assay

Cell viability was measured by plating 4000 cells/well in 96-well plates overnight. The next day the cells were treated with indicated drugs or compounds at indicated concentrations. At indicated time points, cell viability was measured according to the manufacturer’s instructions. The assay is based on measurement of a conserved and constitutive protease activity within live cells, and therefore, serves as a biomarker of cell viability. The live-cell protease activity is restricted to intact viable cells and is measured using a fluorogenic, cell-permeant, peptide substrate (Gly-Phe-AFC). The substrate enters intact cells, where it is cleaved by the live-cell protease activity to generate a fluorescent signal proportional to the number of living cells. The data is presented as percent against control, which was taken as 100% viable.

### CyQUANT NF Cell Proliferation Assay

A2780 and C200 cells (4000 cells/well) were plated in 96-well plates in glucose free Roswell Park Memorial Institute 1640 media containing 5% dialyzed fetal bovine serum supplemented with (i) no glucose (glucose−), (ii) 10 mM glucose (glucose+), and (iii) 1 mM sodium pyruvate for 48 h. Proliferation of A2780 and C200 cells was measured at various time points using the CYQUANT® NF Cell Proliferation Assay kit (Thermo Fisher Scientific) and measuring fluorescence intensity with excitation at 485 nm and emission detection at 530 nm according to the manufacturer’s instructions.

### Western blots

Cells were plated and lysed at 80% confluency using lysis buffer (50 mM Tris-HCl, pH 7.5, 250 mM NaCl, 5 mM ethylenediaminetetraacetic acid, 50 mM NaF, and 0.5% Nonidet P-40) containing a protease inhibitor cocktail (Sigma). Equal amounts of isolated protein (50 µg) measured by the Bradford method (Bio-Rad) was resolved by sodium dodecyl sulfate polyacrylamide gel electrophoresis and transferred to a nitrocellulose membrane. The blots were blocked for 1 h at room temperature in a 5% dry milk and TBST solution followed by incubation overnight at 4 °C with specific primary antibodies (lactate dehydrogenase-α, PGCα, Cox Vb, and GLUT1 antibodies purchased from Sigma) in 5% milk and TBST solution. Lastly, blots were probed with their respective secondary antibody and developed^29^.

### Mitochondrial membrane potential

JC-1 (5,5′,6,6′-tetrachloro-1,1′,3,3′-tetraethylbenzimi- dazolylcarbocyanine iodide) staining that differentiates energized mitochondria from de-energized mitochondria by enumerating the normally green fluorescence dye (J-monomers) and its conversion to red fluorescence (J-aggregates) upon accumulation in energized mitochondria was used (Abcam, Cambridge, MA). The higher red to green fluorescence intensity ratio indicated the increased mitochondrial membrane potential. Carbonyl cyanide 3-chlorophenyl hydrazone was used as a positive control.

### Reactive oxygen species (ROS) measurement

ROS was determined using the membrane-permeable fluorescent dye 2′,7′-dichlorofluorescin diacetate (DCFDA) in serum-free medium, as described previously^58^. Cells were treated with 5 µM DCFDA dye and fluorescence was measured at excitation 485 nm and emission 530 nm for various time periods from 10 to 60 minutes using Synergy H1 Hybrid Reader Monochromator System (BioTek, Winooski, VT)^58^ Data shown is read at 30 minutes.

### Fatty acid oxidation

Fatty acid oxidation-linked OCR was measured in the presence of palmitate (30 µM) as a substrate using *Krebs-Henseleit*-bicarbonate medium. Etomoxir was added at a final concentration of 0.1 mM. Fatty acid-linked OCR was measured by sequential addition of oligomycin (3.1 μM), FCCP (4 μM), rotenone (4 μM) and antimycin (2 μM) to the indicated final concentrations. Measurements were performed according to the Seahorse Bioscience protocol for fatty acid oxidation^61^.

### Isolation of ovarian cancer cells from patient specimen

Patient samples were collected at the time of surgery under an approved institutional review board protocol (IRB# 9520) and after obtaining informed patient consent, as per the approved methods, guidelines and regulations of the institutional IRB. Solid tumor from the ovary or other metastatic sites was dissociated using the Miltenyi Biotec Human Tumor Dissociation Kit (Auburn, CA) following the soft tumor dissociation protocol supplied. Ascites was isolated using the protocol established by Shepherd *et al*.^62^. Briefly, ascites and VOSE media supplemented with 15% fetal bovine serum and 1% PennStrep was plated in a 1:1 ration into T75 flasks. Cells were incubated at 37 °C with 4% carbon dioxide and left undisturbed for 3–5 days. After, cells were washed with phosphate buffered saline and given fresh media or frozen dependent upon the confluence of each sample. Once cells reached confluency, they were subjected to Seahorse analysis and cisplatin toxicity assay by MTT as described earlier^55^. **Use of human participants:** The collection and subsequent studies with human material was approved by the institutional Henry Ford Hospital IRB committee after a full board review. Specimen were collected after informed patient consent at the time of the surgery. Before sending the specimen to the laboratory they were de-identified and coded. We have used no data or images that can identify the participant in any manner. We confirming that all experiments were performed in accordance with relevant guidelines and regulations.

### Data and statistical analysis

Data were plotted and statistically analyzed using two-tailed *t*-tests or unpaired *t*-tests (GraphPad Prism Software, La Jolla, CA). Each experiment was performed a minimum of 3 times.

### Data Availability

All data generated or analyzed during this study are included in this published article (and its Supplementary Information files). We will make all materials, data and associated protocols promptly available to readers without undue qualifications in material transfer agreements (MTA) on request to the corresponding author. The only exception will be the cell lines obtained under MTA (A2780 and C200). No datasets were generated or analyzed during the current study.

## Electronic supplementary material

Supplementary Information
