# Supplementary material for: Bioenergetic Adaptations in Chemoresistant Ovarian Cancer Cells
Source: Sci Rep. 2017 Aug 18;7:8760. doi: 10.1038/s41598-017-09206-0 (PMC5562731; doi:10.1038/s41598-017-09206-0)

SUPPLEMENTARY INFORMATION

Bioenergetic adaptations in chemoresistant Ovarian Cancer Cells

Sajad Dar[1†] , Jasdeep Chhina[1†], Ismail Mert[2], Dhananjay Chitale[3], Thomas Buekers[1], Hareena Kaur[1], Shailendra Giri[4], Adnan Munkarah[1] & Ramandeep Rattan[1*]

[1]Division of Gynecology Oncology, Department of Women's Health Services, Henry Ford Health System, Detroit, MI 48202, USA

[2]Department of Obstetrics and Gynecology, Wayne State School of Medicine, Detroit, MI 48202, USA

[3]Department of Pathology, Henry Ford Health System, Detroit, MI 48202, USA

[4]Department of Neurology, Henry Ford Health System, Detroit, MI 48202, USA

[†]These authors contributed equally to the work.

Supplementary Figure 1: Bioenergetics profile of ovarian cancer cell lines. (A) Basal ECAR represents the glycolysis measure at resting state, (B) glycolytic capacity represents the total ability of the cell to perform glycolysis (resting state and enforced combined) and (C) glycolytic reserve defines the capacity to increase glycolysis when mitochondria is compromised, and these were calculated as described in the Materials and Methods. (D) Basal OCR represents the mitochondrial respiration at resting state, (E) maximum respiration indicates the cellular reaction to an increased ATP demand using FCCP mitochondrial uncoupler, and (F) respiratory reserve and ATP-linked respiration indicate greater capacity to utilize mitochondria. These were calculated as described in the Materials and Methods. Abbreviations: OCR: Oxygen Consumption Rate; ECAR: Extracellular Acidification Rate; ATP: adenosine triphosphatase; FCCP: carbonylcyanide-p-trifluoromethoxyphenyl hydrazine

Supplementary Figure 2: Fatty acid oxidation linked oxygen consumption rate. Representative fatty acid oxidation linked OCR in chemosensitive A2780 (A) and PEO1 (B) and their respective chemoresistant counterparts C200 (C) and PEO4 (D). BSA represents the basal rate that is induced by addition of palmitate. Inhibition by etoximir indicates that the OCR is fatty acid derived. Abbreviations: BSA: Bovine Serum Albumin; OCR: Oxygen Consumption Rate.

Supplementary Figure 3: Taxol does not induce any bioenergetics change in chemosensitive cells. A2780 chemosensitive cells were treated with a non-toxic low dose of taxol (1nM) for 24 and 48 hours and then subjected to measurement of (A) ECAR profile after (1) glucose (fuel for glycolysis), (2) oligomycin (an ATP synthase blocker) and (3) 2DG (an inhibitor of glycolysis) injections (C) OCR profile measured under similar conditions after (1) oligomycin (ATP synthase inhibitor), (2) FCCP (an electron transport chain uncoupler) and (3) rotenone (an

inhibitor of electron transport chain) injections. (B) Basal glycolysis and (D) Basal mitochondrial respiration was unchanged by taxol exposure. All Seahorse experiments were carried out in triplicates and replicated thrice. Abbreviations: ATP: adenosine triphosphatase; 2DG: 2-deoxyglucose; FCCP: carbonylcyanide-p-trifluoromethoxyphenyl hydrazine. OCR: Oxygen Consumption Rate; ECAR: Extracellular Acidification Rate.

Supplementary Figure 4: Representative original untouched images of western blot. The protein extracted from cells was subjected to western blot and immuno-blotted for expression of PGC1α, CoxVb, GLUT1 and LDHa. Alpha- tublin acted as the loading control. Blots are representative of 2 individually performed experiments. A low exposure and a high exposure image is included. Abbreviations: PGC1α: peroxisome proliferator-activated receptor gamma coactivator 1-alpha; CoxVb: cytochrome c oxidase subunit 5B; GLUT1: glucose transporter 1 and LDHa: lactate dehydrogenase-A.

FIGURE S1

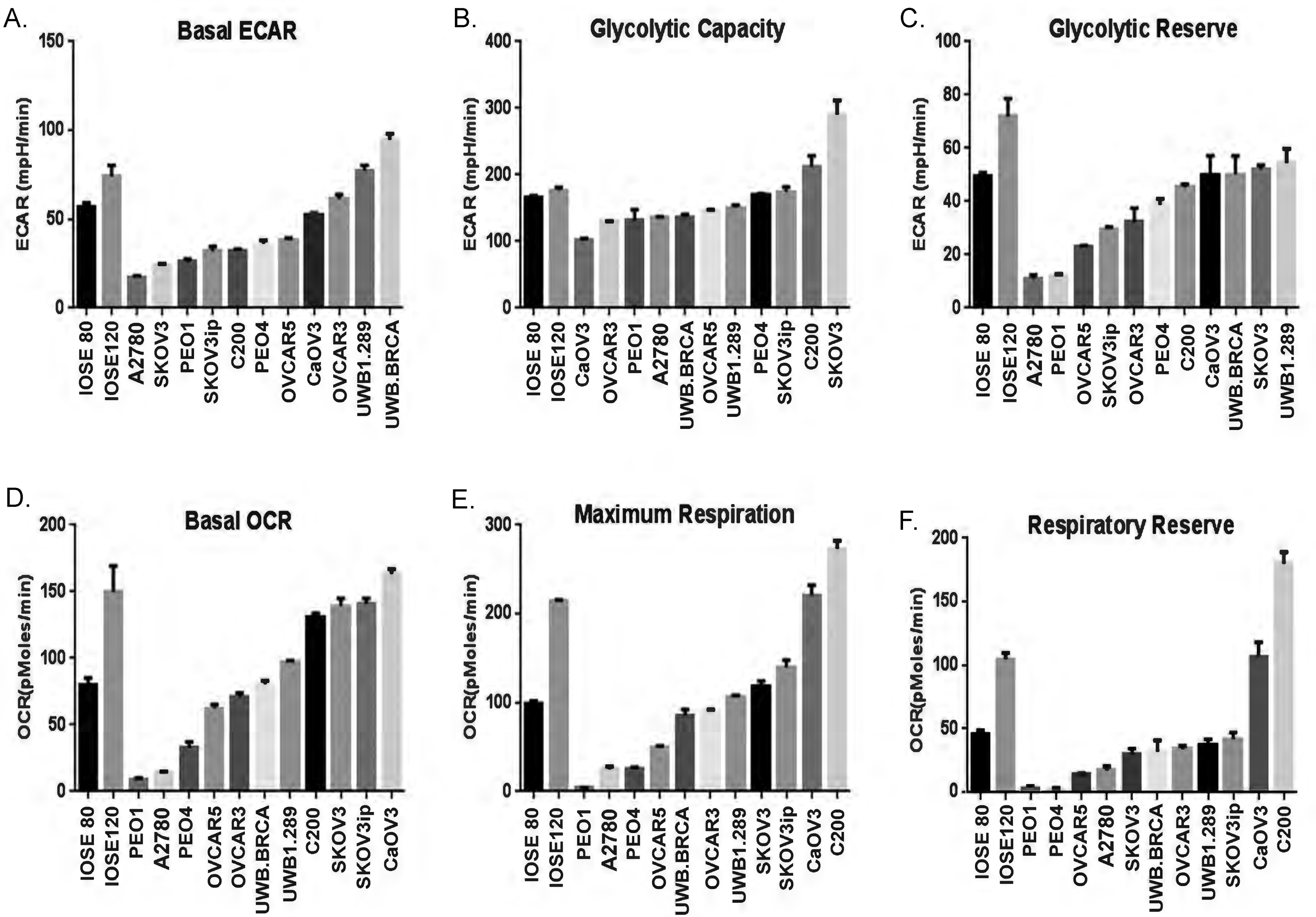

A.
Basal ECAR
ECAR (mpH/min)
0
50
100
150
IOSE 80
IOSE120
A2780
SKOV3
PEO1
SKOV3ip
C200
PEO4
OVCAR5
CaOV3
OVCAR3
UWB1.289
UWB.BRCA
B.
Glycolytic Capacity
ECAR (mpH/min)
0
100
200
300
400
IOSE 80
IOSE120
CaOV3
OVCAR3
PEO1
A2780
UWB.BRCA
OVCAR5
UWB1.289
PEO4
SKOV3ip
C200
SKOV3
C.
Glycolytic Reserve
ECAR (mpH/min)
0
20
40
60
80
100
IOSE 80
IOSE120
A2780
PEO1
OVCAR5
SKOV3ip
OVCAR3
PEO4
C200
CaOV3
UWB.BRCA
SKOV3
UWB1.289
D.
Basal OCR
OCR(pMoles/min)
0
50
100
150
200
IOSE 80
IOSE120
PEO1
A2780
PEO4
OVCAR5
OVCAR3
UWB.BRCA
UWB1.289
C200
SKOV3
SKOV3ip
CaOV3
E.
Maximum Respiration
OCR(pMoles/min)
0
100
200
300
IOSE 80
IOSE120
PEO1
A2780
PEO4
OVCAR5
UWB.BRCA
OVCAR3
UWB1.289
SKOV3
SKOV3ip
CaOV3
C200
F.
Respiratory Reserve
OCR(pMoles/min)
0
50
100
150
200
IOSE 80
IOSE120
PEO1
PEO4
OVCAR5
A2780
SKOV3
UWB.BRCA
OVCAR3
UWB1.289
SKOV3ip
CaOV3
C200

FIGURE S2

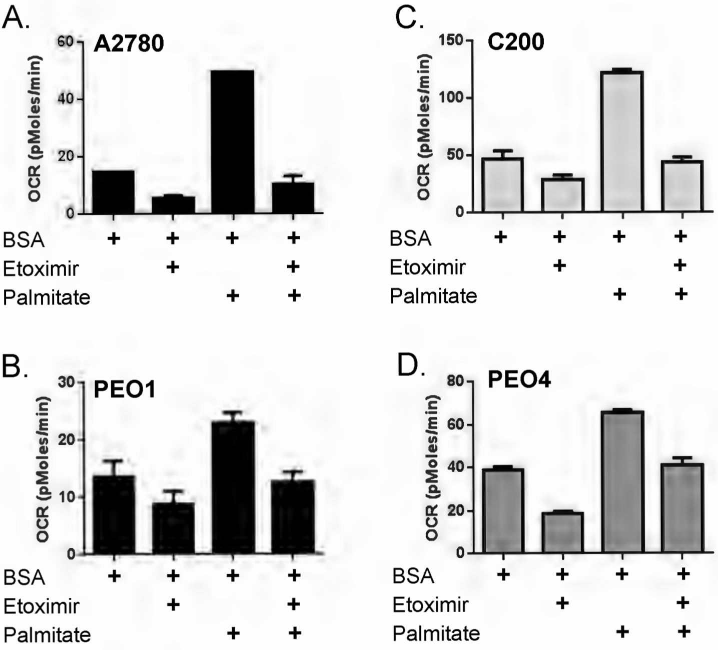
A.
A2780
OCR (pMoles/min)
60
40
20
0
BSA + + + +
Etoximir + +
Palmitate + +
B.
PEO1
OCR (pMoles/min)
30
20
10
0
BSA + + + +
Etoximir + +
Palmitate + +
C.
C200
OCR (pMoles/min)
150
100
50
0
BSA + + + +
Etoximir + +
Palmitate + +
D.
PEO4
OCR (pMoles/min)
80
60
40
20
0
BSA + + + +
Etoximir + +
Palmitate + +

FIGURE S3

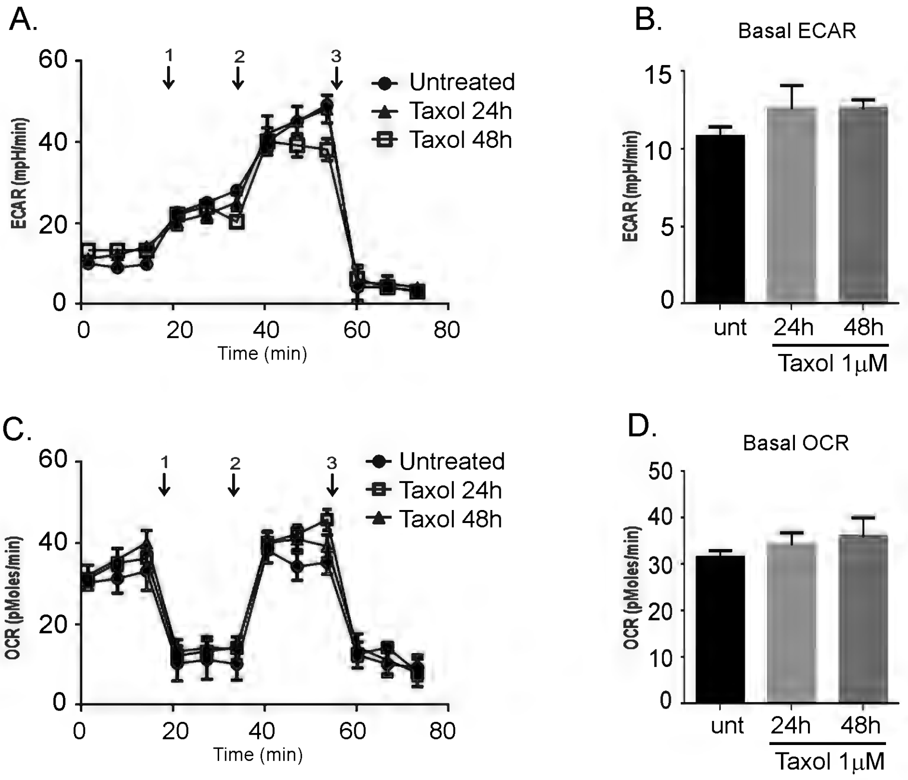
A.
60
40
20
0
ECAR (mpH/min)
1
2
3
Untreated
Taxol 24h
Taxol 48h
0
20
40
60
80
Time (min)
B.
Basal ECAR
15
10
5
0
ECAR (mpH/min)
unt
24h
48h
Taxol 1μM
C.
60
40
20
0
OCR (pMoles/min)
1
2
3
Untreated
Taxol 24h
Taxol 48h
0
20
40
60
80
Time (min)
D.
Basal OCR
50
40
30
20
10
0
OCR (pMoles/min)
unt
24h
48h
Taxol 1μM

FIGURE S4

LOW EXPOSURE

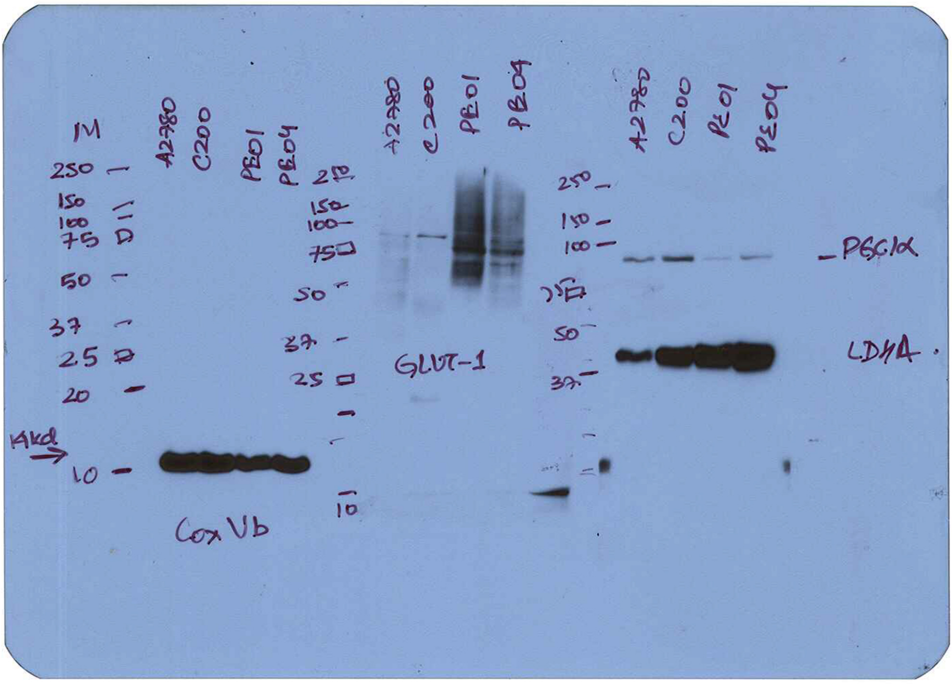

HIGH EXPOSURE

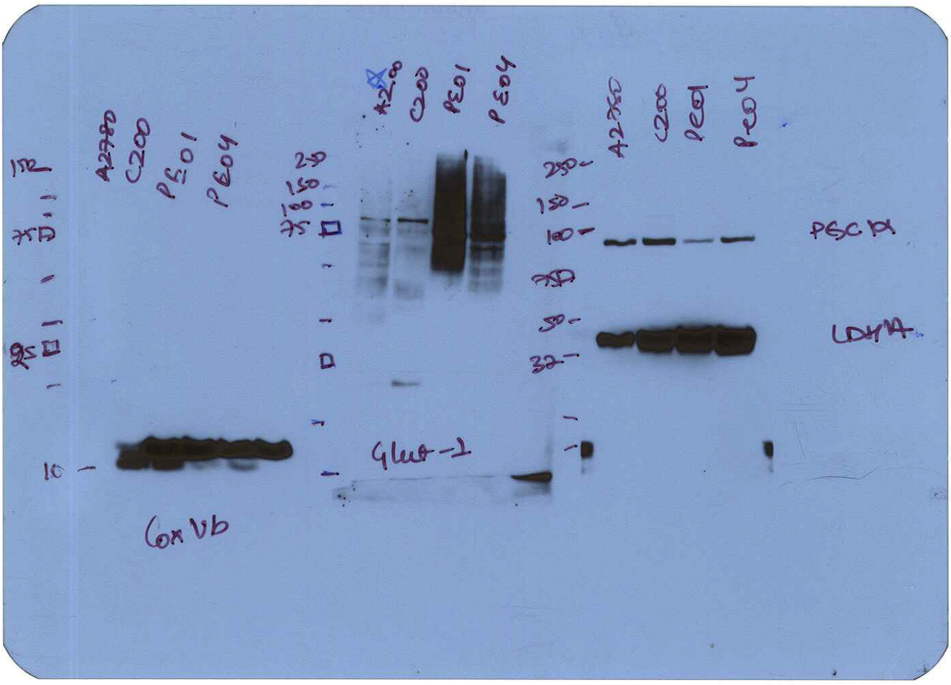

TUBULIN

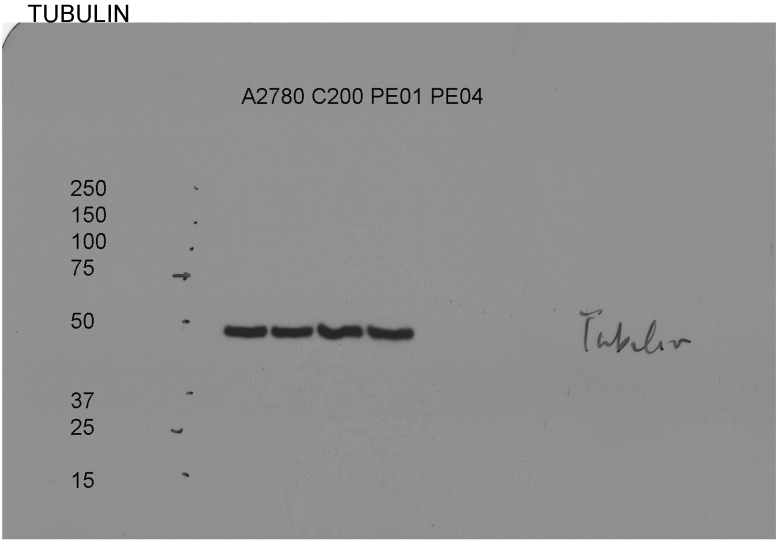

Supplement: Supplementary file 1 — Supplementary Information [file 41598_2017_9206_MOESM1_ESM.pdf]
